# A Case Report of a Young Patient Presenting With Syncope Secondary to Arrhythmogenic Ventricular Cardiomyopathy

**DOI:** 10.7759/cureus.33731

**Published:** 2023-01-13

**Authors:** Zahid Khan, Tom Rayner, Dinesh Sethumadhavan, Sahar Hamid

**Affiliations:** 1 Acute Medicine, Mid and South Essex NHS Foundation Trust, Southend on Sea, GBR; 2 Cardiology, Bart’s Heart UK, London, GBR; 3 Cardiology and General Medicine, Barking, Havering and Redbridge University Hospitals NHS Trust, London, GBR; 4 Cardiology, Royal Free Hospital, London, GBR; 5 Cardiology, Bart's Heart Centre, London, GBR; 6 Internal Medicine, Barking, Havering and Redbridge University Hospitals NHS Trust, London, GBR

**Keywords:** right bundle branch block, cardioinhibitory syncope, prevention of syncope, implantable cardioverter-defibrillator (icd), emergency echocardiography, cardiac magnetic resonanced imaging, ventricular dysrhythmia, life-threatening arrhythmia, arrhythmogenic right ventricular cardiomyopathy (arvc/d), arrhythmogenic cardiomyopathy

## Abstract

Arrhythmogenic ventricular cardiomyopathy is an inherited condition mainly affecting adults. A 35-year-old Asian male patient presented with syncope while walking home. He experienced a number of episodes of light-headedness and dizziness over the past few weeks. A clinical examination found visible injuries to his face and hands. An electrocardiogram showed right axis deviation and right bundle branch block. Echocardiography showed normal biventricular function and the left ventricular ejection fraction was > 55%. A computerized tomography scan of the head and face showed a small fracture to the superior maxillary wall and a computerized tomography pulmonary angiogram demonstrated an inflammatory nodule with right upper and middle lobes changes as well as right hilar lymphadenopathy, suggestive of possible tuberculosis. Blood tests were unremarkable, and troponin was negative. Cardiovascular magnetic resonance imaging showed preserved biventricular function, mild bi-atrial dilatation, and extensive, crescentic-shaped, subepicardial late gadolinium enhancement from basal to apical inferior, basal to apical lateral, and mid to apical anterior segments of the left ventricle suggestive of arrhythmogenic ventricular cardiomyopathy. The patient was commenced on bisoprolol and had an implantable cardioverter defibrillator fitted. He was discharged home with outpatient cardiology follow-up.

## Introduction

Arrhythmogenic ventricular cardiomyopathy (AVC) is an inherited condition, first described by Frank and Fontain in 1978 [1]. They described it as characterized by total or partial replacement of right ventricular muscle with fibro-fatty tissue. The fibro-fatty replacement of the myocardium increases the risk of arrhythmias in patients with AVC. AVC can present as arrhythmogenic right ventricular cardiomyopathy (ARVC), arrhythmogenic left ventricular cardiomyopathy (ALVC), or biventricular cardiomyopathy. The incidence of arrhythmogenic right ventricular cardiomyopathy (ARVC) is estimated to vary from 1 in 1000 to 1 in 1250 people [2]. Most younger patients do not have any symptoms and may only be diagnosed when they present with a syncopal episode or sudden cardiac arrest [2]. The commonest cause for fibrous fat replacement of myocardium in patients with ARVC is mutations in genes encoding desmosomal proteins, such as the desmoglein-2 gene (DSG2) [3]. Other genes found to have alterations in patients with ARVC include plakoglobin (JUP), desmocollin 2 (DSC2), plakophilin 2 (PKP2), and desmoplasia (DSP) [3]. It is most commonly diagnosed on post-mortem examination following the death of an individual; however, younger patients may also present with sustained ventricular tachycardia, left-sided, right-sided, or biventricular failure [1]. There is a significant geographical variation in the distribution of the disease; for example, it is the most common cause of death in young individuals below 35 years of age and young athletes in Italy whereas it accounts for about 5% of sudden cardiac death in individuals < 65 and 3-4% in young athletes [4]. ARVC most commonly affects male patients and about 80% of individuals diagnosed with ARVC are < 40 years old [4]. It should commonly be suspected in individuals presenting with cardiac arrest, syncopal episodes, or arrhythmias and is commonly seen in patients with hypertrophic obstructive cardiomyopathy (HOCM) [1,4].

## Case presentation

**Figure 1:**
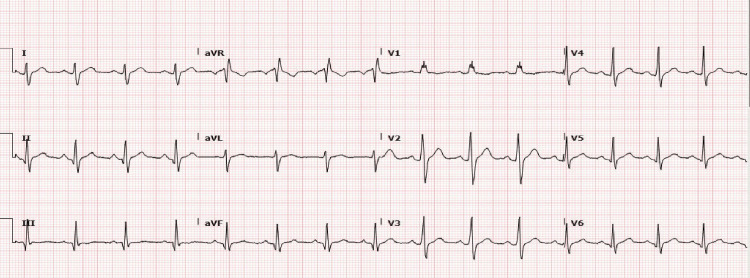
Electrocardiogram showing normal sinus rhythm with right axis deviation and right bundle branch block

A 35-year-old Asian male presented to the hospital with a syncopal episode while he was walking home from a restaurant. He had two glasses of wine that evening but had been feeling dizzy and lightheaded in recent weeks. He did not have any significant past medical history and was not on any regular medication. He denied any palpitations, chest pain, or shortness of breath. He could not recall the syncopal episode fully and was found at the roadside by bystanders. On arrival at the hospital, his electrocardiogram (ECG) showed normal sinus rhythm (NSR) with right axis deviation (RAD) and right bundle branch block (RBBB) (Figure 1).

**Figure 2:**
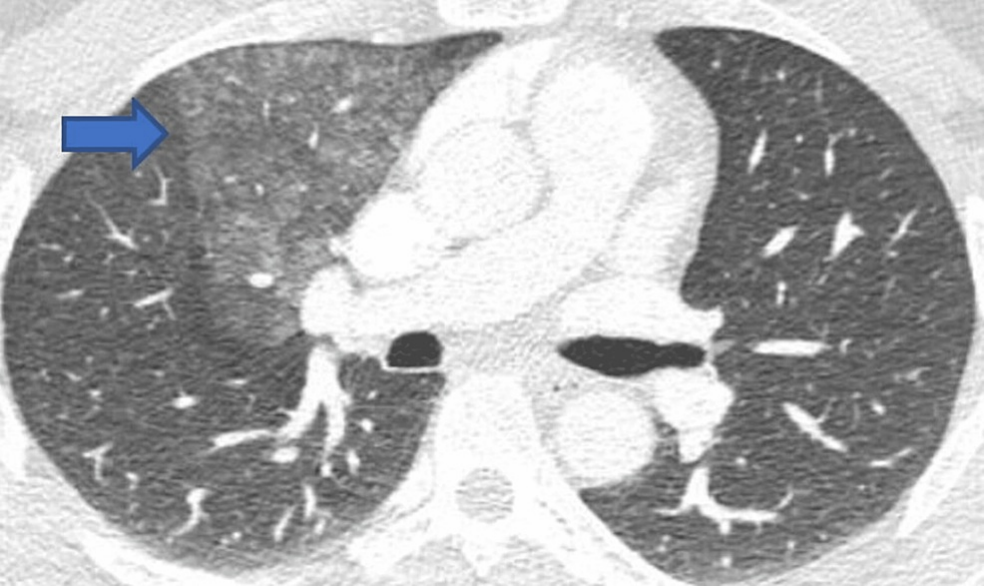
Computerized tomography pulmonary angiogram showing centrilobular nodular opacification affecting the right upper and middle lobes as shown by the blue arrow.

He had sustained facial and hand injuries. Most blood tests including troponin T, creatinine kinase, HbA1c, immune screening tests such as complement levels, rheumatoid factor, proteinase 3 antibody, myeloperoxidase antibody, myocardial antibody, and cardiolipin antibody, thyroid function tests, creatinine kinase were within normal limits. Viral screening for human immunodeficiency virus, hepatitis, and COVID-19 was negative. The erythrocyte sedimentation rate was mildly elevated at 14 mm/hour (Normal range: 1-10 mm/hr). Computerized tomography (CT) scan of the head and face showed a small fracture to the superior maxillary wall with overlying soft tissue swelling without any intracranial pathology. CT pulmonary angiogram showed florid centrilobular nodular opacification affecting the right upper and middle lobes, hilar lymphadenopathy, and anterior abdominal lymphadenopathy suggestive of possible tuberculosis (Figure 2). Following further investigation and respiratory input, it was felt tuberculosis was highly unlikely. He was commenced on bisoprolol 2.5 mg once daily.

**Figure 3:**
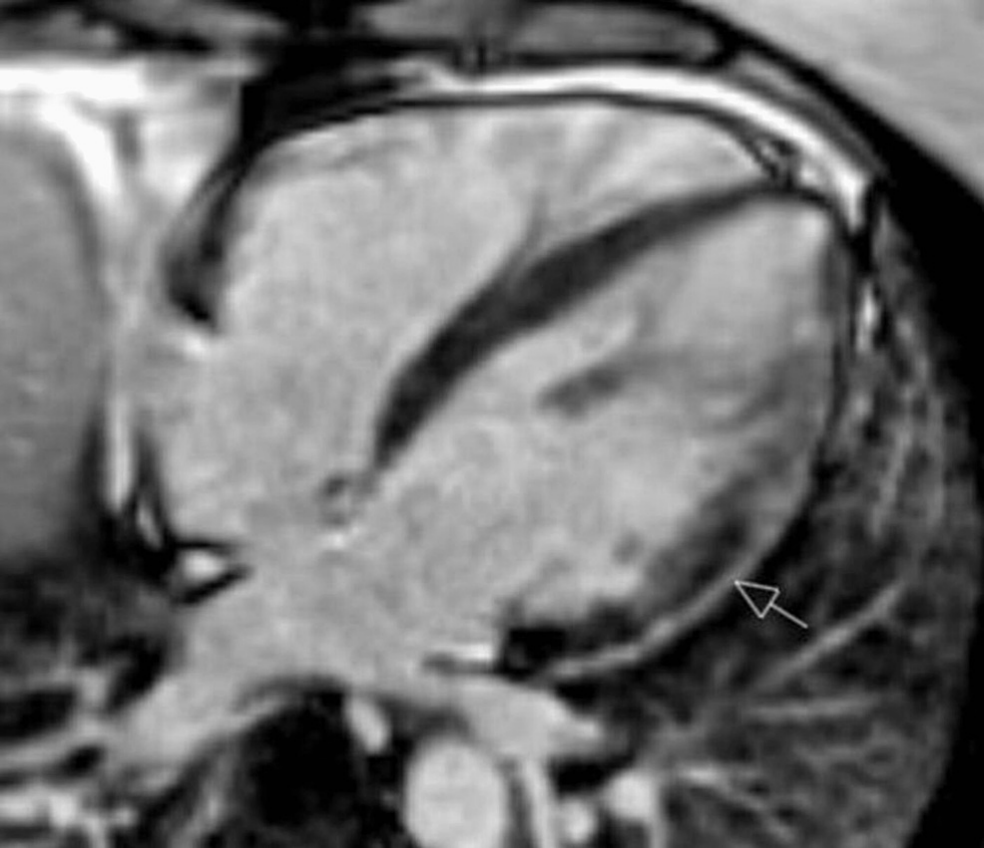
Cardiovascular magnetic resonance imaging showing extensive crescentic-shaped subepicardial late gadolinium enhancement as shown by the arrow.

**Figure 4:**
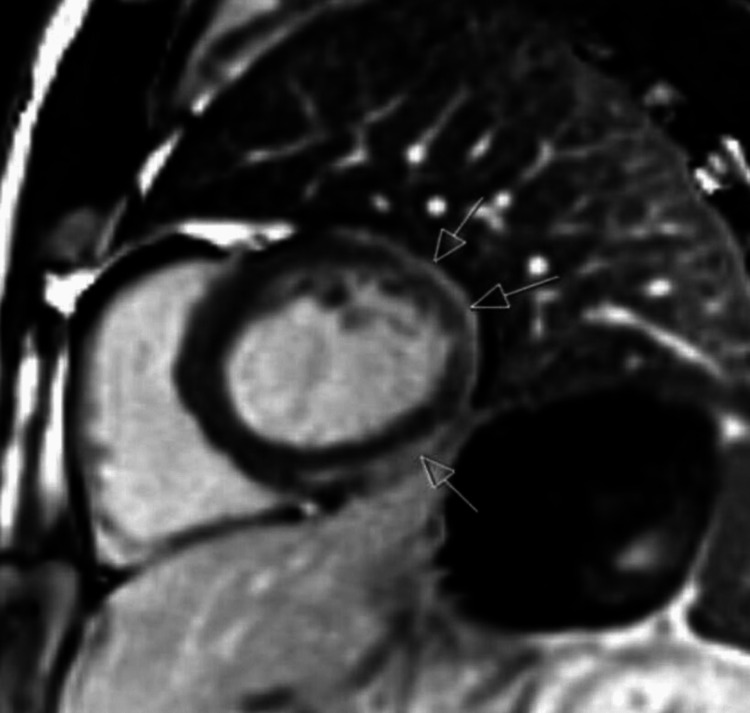
Cardiovascular magnetic resonance imaging (CMR) short axis view showing extensive, crescentic-shaped, subepicardial late gadolinium enhancement from basal to apical inferior, basal to apical lateral, and mid to apical anterior segments as shown by arrows.

**Figure 5:**
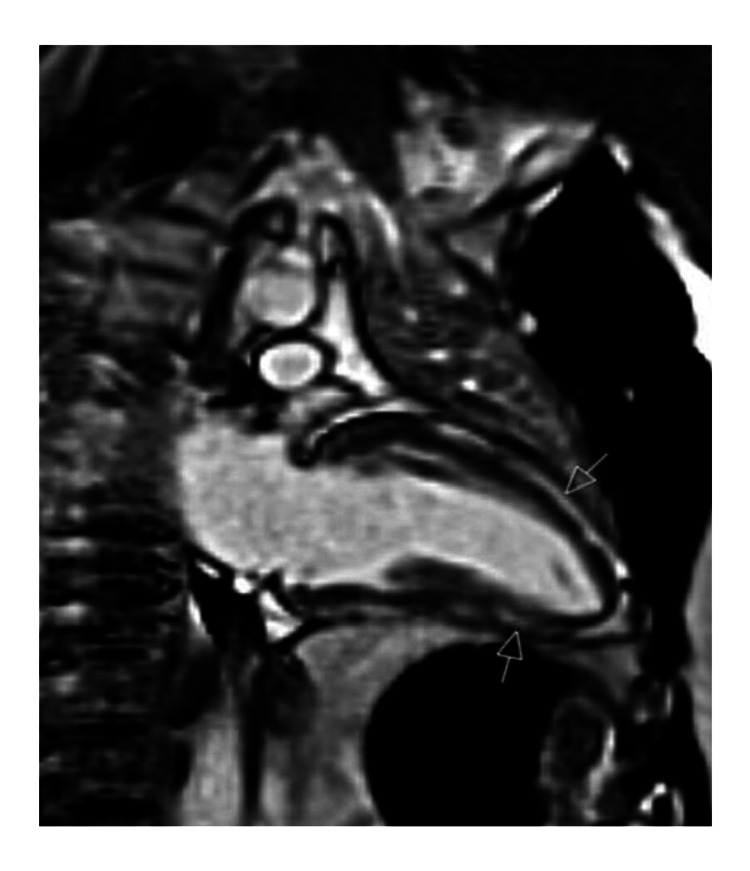
Cardiovascular magnetic resonance imaging of two-chamber view showing extensive, crescentic-shaped, subepicardial late gadolinium enhancement as shown by arrows.

Echocardiography showed normal biventricular function with an estimated left ventricular ejection fraction (LVEF) of 55% and no regional wall motion abnormalities (Videos 1-2). Cardiovascular magnetic resonance imaging (CMR) showed normal biventricular function, mild atrial dilatation, and extensive, crescent-shaped, subepicardial late gadolinium enhancement seen in basal to apical inferior, basal to apical lateral, and mid to apical anterior segments (Figures 3-5).

**Video 1:** Echocardiography of parasternal long axis view showing normal left ventricular function.

**Video 2:** Echocardiography of apical four-chamber view showing good biventricular function.

The patient was offered an implantable cardioverter defibrillator (ICD) for secondary prevention. The patient was discussed in a multidisciplinary team meeting (MDT) for subcutaneous versus transvenous ICD and had subcutaneous ICD screening which was normal. A transvenous ICD was implanted following a multidisciplinary team meeting. Chest radiography post-ICD implantation showed satisfactory leads positioning (Figure 5). Post-implantation device checks were satisfactory, and the patient was discharged home on bisoprolol 2.5 mg once daily with outpatient cardiology follow-up in the device clinic.

**Figure 6:**
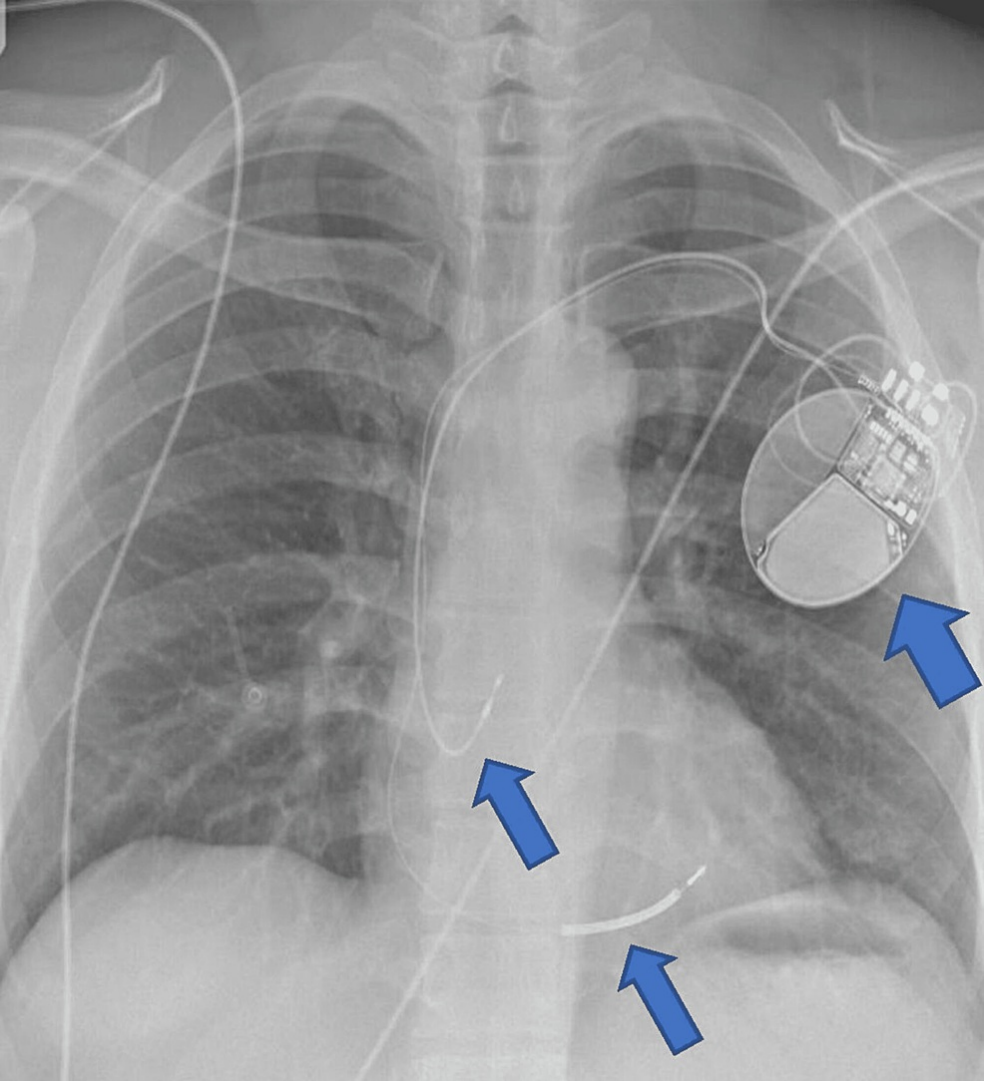
Post-ICD implantation chest radiography ICD: implantable cardioverter defibrillator

## Discussion

ARVC is a rare inherited cardiomyopathy, and the reported incidence varies from 1:1000 to 1:1250 in some studies and 1:1000 to 1:5000 in other studies [2,3]. ARVC is characterized pathologically by fibrofatty replacement of the right ventricular myocardium and clinically by ventricular tachycardia that can result in sudden cardiac death, mostly in young patients and athletes whereas ALVC patients have fibrofatty myocardial replacement affecting the subepicardial layers in the left ventricle [5]. The clinical findings and morphological features of the disease support the non-ischaemic atrophy of the right ventricular myocardium rather than the congenital absence of the myocardium that leads to arrhythmias in adolescents and young adults [6,7]. AVC is the second leading cause of death in young patients following hypertrophic cardiomyopathy [1]. Thiene et al. (1988) reported autopsy findings suggestive of AVC in a series of cases of exertional sudden cardiac deaths in young adults; the ECGs in these patients demonstrated right precordial leads with negative T waves and left bundle branch (LBBB) morphology type ventricular arrhythmia [8]. This makes sense as the arrhythmia generally originates from the right ventricle and therefore most patients have LBBB morphology on ECGs. Though about half of the AVC cases are familial in origin and are inherited in an autosomal dominant pattern, these cases rarely demonstrate autosomal recessive inheritance [2]. The fibrofatty replacement of the myocardium is mainly transmural and accounts for aneurysmal dilations of the diaphragmatic, apical, and infundibular regions in almost 50% of cases, also known as the "triangle of dysplasia" [8,9].

Several case reports of patients with ARVC have been published over the last few years. Khan et al. (2022) reported a case of a 62-year-old patient who had two out-of-hospital cardiac arrests in a span of a decade and was successfully resuscitated both times. His coronary arteries were unobstructed and CMR findings were suggestive of ARVC, for which he had an ICD implanted for primary prevention [1]. Latt et al. (2017) published a case report of a 33-year-old male athlete presenting with palpitations and chest pain, on a background of similar episodes five years previously [10,11]. His ECG showed diffuse symmetric T waves in precordial leads. The echocardiogram was normal. His Holter monitor revealed frequent premature ventricular ectopic beats and frequent episodes of non-sustained ventricular tachycardia with LBBB. CMRI showed features of ARVC, with a dyskinetic right ventricle (RV) and foci of fat in the RV side of the interventricular septum. The authors were able to induce VT with LBBB, with the substrate in the RV during an electrophysiology study. Following this, he underwent ICD insertion. An echocardiogram is recommended for all patients and family members with suspected cases of AVC and can be useful to assess the progression of the disease [5].

Patients with AVC may present with biventricular failure, isolated right, or left-sided heart failure in their fourth or fifth decade due to dilatation and thinning of both ventricles, right or left ventricle alone, and frequent ventricular ectopics [4, 11]. In 2020, international experts updated the original task force criteria and provided updated diagnostic criteria known as "The Padua Criteria" developed from the diagnostic approach to AVC over the past 30 years [12]. These criteria were reviewed and agreed upon by various international experts and have become an international consensus document to diagnose AVC. This new classification unlike the previous document incorporated the phenotype of AVC such as the "dominant right" variant also known as ARVC with no LV abnormalities, the "biventricular disease" variant that affects both LV and RV, and the "dominant left" variant characterized by LV involvement only [12]. CMR may show LGE involving the whole LV free wall and anterior septum in ALVC, extending from basal to apical regions also known as a "ring-like" pattern [12]. AVC can also be a manifestation of inflammatory diseases such as myocarditis [12, 13]. This patient had extensive LGE in the subepicardial region and inverted T waves in V4-V6. According to the 2020 ALVC consensus document, subepicardial or mid myocardial LGE in ≥ 1 Bull's eye segment is a major criterion and T waves inversion in the left precordial leads in absence of left bundle branch block is a minor criterion [12]. Similarly, small QRS complexes in limb leads in absence of obesity, emphysema, pericardial effusion, and frequent ventricular extrasystole >500 per 24 hours, and non-sustained or sustained VT in presence of RBBB are also minor criteria. Our patient also had a few ventricular ectopics during admission and was suspected to have arrhythmia leading to a syncopal episode. It is important to mention here that the diagnosis of ALVC cannot be achieved on the basis of LV phenotypic criteria only as there is significant overlap in the phenotypic features of AVC with other conditions such as myocarditis, sarcoidosis, and dilated cardiomyopathy (DCM). The diagnosis of ALVC therefore, in addition to LV phenotypic features also requires demonstration of a positive genotyping for pathogenic or likely pathogenic ACM‐causing gene mutations [12, 13]. The CMR in our patient did not show any active inflammation; however, myocarditis is one potential differential diagnosis in this patient which could present with LGE and scarring in very severe forms.

## Conclusions

In conclusion, AVC can be right-sided, left-sided, or biventricular and is a common cause of death in younger and middle-aged patients, particularly athletes. AVC can be extremely challenging to diagnose in certain cases due to the overlapping phenotypic features with other diseases such as myocarditis. Most patients have fatal arrhythmias and present with cardiac arrest or syncopal episodes. Patients with arrhythmogenic ventricular cardiomyopathy should have an ICD implanted to minimize risk of sudden cardiac death. It is more common in males and young athletes and most cases are diagnosed on post-mortem examination. We feel it is essential that clinicians are aware of AVC, and consider this diagnosis when patients present with syncopal episodes or new ventricular arrythmia.
